# Structure of microemulsions in the continuous phase channel

**DOI:** 10.1140/epje/s10189-023-00337-z

**Published:** 2023-09-05

**Authors:** Robert Franz Schmidt, Sylvain Prévost, Michael Gradzielski, Thomas Zemb

**Affiliations:** 1 Stranski-Laboratorium Für Physikalische Und Theoretische Chemie, Institut Für Chemie, Technische Universität Berlin, Straße Des 17. Juni 124, 10623 Berlin, Germany; 2 Institut Laue-Langevin, 71 Avenue Des Martyrs CS 20156, 38042 Grenoble Cedex 9, France; 3 UMR 5257 - CEA/CNRS/UM/ENSCM, Institut de Chimie Séparative de Marcoule, ICSM, 30207 Marcoule, France

## Abstract

We have studied the microemulsion and lamellar phases of two of the most commonly described systems based on nonionic C_12_E_5_ and ionic AOT surfactants. We show that C_12_E_5_ is best described by the symmetric disordered open connected lamellar model (DOC-lamellar), contrary to the more commonly employed standard flexible model. In the case of AOT, the bicontinuous microemulsion structure is best described by the standard flexible model at high temperatures. Around room temperature, connected cylinders in a molten cubic crystal phase are the only description which corresponds to the data. In the lamellar phase, around one third of the available surface area is lost in fluctuations and defects. Comparing structurally predictive models with results from conductivity measurements show that salt adsorption in the hydrated ethoxy groups is dominant for C_12_E_5_ (nonionic). For AOT, our conductivity measurements clarify the role of tortuosity versus cation absorption.

**Supplementary Information:**

The online version contains supplementary material available at 10.1140/epje/s10189-023-00337-z

## Introduction

Microemulsions have a long time ago moved from a laboratory curiosity to a class of self-assembled systems widely used in applications [1]. Structurally, their extreme cases are oil-in-water (O/W) droplets, water-in-oil (W/O) droplets, and bicontinuous systems with a sponge structure. Droplet systems are typically encountered in cases of a large excess of one component. One of the most intriguing structural properties of microemulsions is their ability to become inverted from O/W to W/O without ever observing a phase transition during this profound structural change. This means that the curvature of the amphiphilic film can be inverted in a fully continuous way without reaching a situation with a negative curvature of the free energy as a function of composition, which would result in separation into two phases. Moreover, the notion of curvature alone is only applicable for interfacial film thicknesses that are negligible versus curvature [2]. For this reason, systems for which the ternary phase diagram is completely symmetric vs. volume fraction are very rare [3].

Considering the molecular film thickness allows to add bending Gibbs energy to the hydration energy of ions and head groups [4]. If this is not the case, the frustration of packing must be considered, and a molecular value of the bending constant in k_b_*T*/molecule should be used instead of geometrical quantities related to average and Gaussian curvature of an infinitely thin film in order to be added to other terms of the Gibbs energy of the system [4]. To the best of our knowledge, only one description of a microemulsion in the literature meets this restriction to a surfactant volume fraction less than 1% [3]. All the samples analysed here are at volume fractions of surfactant exceeding this limit, so the molecular bending constant and not the common “Helfrich” expansion is relevant [5].

For nonionic surfactants with ethylene oxide (EO) groups, the system parameter to tune the packing in a continuous transition can simply be the temperature (and changing surfactant concentration [6]), especially for roughly equal amounts of oil and polar, i.e. water plus head groups. More generally, this transition can proceed by changing the oil-to-water ratio and, at the same time, changing temperature [7, 8]. For ionic surfactants, this can also be achieved by changing salinity instead of temperature [9] or by reducing an intrinsically favoured high curvature of an ionic surfactant by high salinity and thereby rendering it sensitive to structural changes induced by temperature variation. In all cases, the equilibrium microstructure of a microemulsion maximises entropy as well as interfacial film frustration, which arises from the mismatch between effective packing in the sample and spontaneous packing of a surfactant monolayer.

Spherical droplets are obtained in extreme cases, which is effective and spontaneous packing match, and are expected at the emulsification failure, where a droplet is swollen to the maximum. Balanced (symmetric) bicontinuous structures are obtained with volume fraction near 50% and a spontaneous packing close to 1. In most practical cases, any type of intermediate microstructure minimising Gibbs energy is present and exactly such situations might frequently be encountered in microemulsion formulations. For comparable amounts of water and oil, the formation of bicontinuous or more or less percolated structures is expected. In large contrast to the situation of spherical droplets, the description of bicontinuous structures is more challenging. There have been a number of theoretical approaches to describe structure and properties under such conditions, but it is still not really clear which model yields a quantitatively correct description. Unfortunately, in that composition range, the vast majority of scattering experiments on microemulsions has been done for equal volume fractions of oil and water, where all models yield similar predictions. In order to discern the reliability and applicability of different theoretical models, structural features such as the size and spacing of domains must be studied for samples with varying oil-to-water ratio. For the so-called alpha-cut at constant oil/water interfacial area, i.e. constant surfactant concentration, models such as de Gennes-Taupin [10] or the Cates-Andelmann-Safran-Roux [11] predict no shift in the peak position when the spontaneous curvature is varied (ideally achieved by changing the temperature), as area and volume fractions remain constant. In contrast, the DOC model [12] predicts a systematic shift of the peak position.

However, so far these predictions were never tested experimentally. This is the aim of this work, using two of the most studied microemulsion systems for which the spontaneous packing parameter (and hence curvature) can be tuned through temperature. To allow more general statements, we employ surfactant films made of flexible nonionic C_12_E_5_ and rigid, ionic AOT. Both systems have been studied before showing a phase behaviour where the microemulsion phase extends continuously from the water-rich to the oil-rich side [13, 14].

## Experimental

### Sample preparation

Two different microemulsions were prepared based on the two surfactants pentaethylene glycol monododecyl ether (C_12_E_5_, Sigma-Aldrich, 98.9) and dioctyl sulfosuccinate sodium salt (AOT, Sigma-Aldrich, 97.9%). The C_12_E_5_ microemulsions were prepared with octane (Sigma-Aldrich, 99.4%) and D_2_O (Deutero, 99.9% D). The AOT microemulsions were prepared with decane (Sigma-Aldrich, 99.5%) and a 0.4wt.% solution of NaCl (Merck, > 99.5%) in D_2_O. For the two systems C_12_E_5_/n-octane/D_2_O and AOT/n-decane/D2O-brine, five samples each were prepared at different oil volume fractions *α* = *V*_oil_/(*V*_oil_ + *V*_D2O_) of 0.25, 0.375, 0.5, 0.625, and 0.75. All samples were prepared by adding the appropriate amounts of C_12_E_5_ or AOT, n-octane or n-decane (all from Sigma-Aldrich), and D_2_O or D_2_O-brine into 4-mL cylindrical glass vials. The samples were then vortexed for ten seconds (where it should be noted that the samples homogenise quickly) and afterwards left on a tube roller overnight before further experiments were done to absolutely ensure equilibration. Table 1 contains the relevant parameters for the prepared samples.

**Table 1:** Relevant parameters of the ten different microemulsion samples. *ϕ*_apolar_ is the apolar volume fraction, *SLD*_apolar_ and *SLD*_polar_ are the apolar and polar scattering length densities (for their calculation see eq. (S2) in the supporting material), respectively, and *N*_water_/*N*_head_ is the ratio of water molecules to surfactant headgroups

surfactant	*c*(surfactant)/wt.%	*ϕ*_apolar_	*SLD*_apolar_/(10^–6^ Å^−2^)	*SLD*_polar_/(10^–6^ Å^−2^)	*N*_water_/*N*_head_
C12E5	6.89	0.27	− 0.50	6.10	226.9
C12E5	7.13	0.38	− 0.51	6.06	192.0
C12E5	7.21	0.50	− 0.51	5.99	159.9
C12E5	7.27	0.61	− 0.51	5.90	126.0
C12E5	7.28	0.73	− 0.51	5.72	89.3
AOT	11.99	0.30	− 0.46	5.74	133.7
AOT	11.94	0.41	− 0.47	5.74	117.5
AOT	12.00	0.53	− 0.47	5.74	98.0
AOT	11.92	0.64	− 0.48	5.74	78.3
AOT	12.17	0.75	− 0.48	5.74	54.0

### Conductivity

Electric conductivity was measured using a conductometer (SevenCompact S230, Mettler-Toledo GmbH, Giessen, Germany) with a 6-mm conductivity sensor (InLab 752-6 mm, Mettler-Toledo GmbH, Giessen, Germany). The temperature was set using a thermomixer (MKR 13 Cooling Thermomixer, Ditabis, Pforzheim, Germany), allowing for five minutes of equilibration time before the measurement was performed. A reference sample of identical volume containing only water was heated in the same way, and the temperature was checked continuously using a digital thermometer. All temperature values are given with an uncertainty of 0.2 °C. Biphasic samples were shaken prior to measurement. The given values were all collected within the first 30 s after shaking before phase separation occurs. The AOT samples have a high base conductivity due to the ionic head group since the aqueous phase is prepared with brine (0.4 wt.% NaCl in D_2_O). For the conductivity measurements of the C_12_E_5_ samples, a 5 mM NaCl solution in D_2_O was used instead of pure D_2_O to increase the base conductivity.

### SANS

The SANS measurements were performed on the D11 instrument at Institut Laue-Langevin in Grenoble, France [15]. A Peltier sample changer was used to accurately set the required temperature. After setting a new temperature, we allowed for five minutes of equilibration time. Each cuvette was then individually shaken and checked before the next measurement was started. The cuvettes were also monitored using a video camera to make sure the samples were homogeneous. For the C_12_E_5_ samples, three sample-to-detector distances of 2.525, 13.524 and 38.024 m were used, covering a total q-range of 1.6 × 10^–3^ to 0.49 Å^−1^. For the AOT samples, two sample-to-detector distances 2.525 and 20.524 m were used, covering a q-range of 3.3 × 10^–3^ to 0.49 Å^−1^. Data reduction was done using GRASP (V.9.20 g) [16].

### Establishing a swelling plot

In ternary systems made from water, oil, and surfactant film, one spectrum measured on absolute scale alone is ambiguous in the determination of the morphology of microstructure. In this case, the determination of the scattering pattern by varying the volume fraction at known interfacial area is necessary to determine microstructures.

It is always possible to fit the scattering data obtained for a microemulsion to any suitable three-parameter expression. A very popular one was introduced by Teubner and Strey (TS) [17]. It allows to derive the domain size *d* and correlation length *ξ* from the peak position and the decay of the scattering intensity. In addition, their combination allows to derive a factor that describes the amphiphilic strength of the surfactant [18]. The TS model is a phenomenological description based on a Ginzburg–Landau expansion, which was subsequently further backed up and improved by a Gaussian random field model (GRFM). The GRFM yields the same scattering expressions but interprets them more directly as a function of the material properties, which depend only on the bending rigidity, temperature, and surfactant concentration [19]. However, it does not take into account the surface imposed by the extensive number of surfactant molecules present at the interface. This model included scattering peak positions that were tested successfully ten years later taking into account the explicit area per molecule, as well as spontaneous curvature [20]. These predictions were tested further by Duvail et al*.* [21] and shown to be close to those from DOC models. The previously unexplained peak shift linked to connections appearing in the structure was predicted using this Gaussian random waves model for the first time [22]. From the dimensionless ratio of correlation length ξ and domain size *d*, the bending rigidity of the membrane can be determined, as successfully shown for SANS data on balanced microemulsions (equal volumes of oil and water) [23].

More recently, MC simulations of dynamically triangulated surfaces of variable topology confirmed the correctness of these theoretical results and showed that the ratio of domain size to correlation length is well described by a combination of logarithmically renormalised bending rigidity and saddle-splay modulus with universal prefactors [24].

In cases where data are analysed in absolute scale over a large dynamic range and fitted also on log scale, a five-parameter expression reflecting area of contact as well as effective curvature is needed in order to obtain a complete description of the scattering curves [25]. This model also contains the Kirste-Porod correction (Kirste-Porod was tested on our experimental data; see Figure S4 in the supporting material) [26]. Again, fitting one spectrum allows to derive quantities qualitatively, but does not yield complete structural information about the type of microstructure. In three-dimensional space, any microstructure can be described as locally globular (polydisperse spheres), locally cylindrical (cylindrical parts connecting globules), or locally lamellar structures (sometime called swollen sponge phases). To represent real cases, Voronoi polyhedral need to be constructed [27]. The peak observed in the scattering structure factor corresponds to the most probable distance between Voronoi polyhedral centres [28]. When the packing term is dominant in the free energy, microstructure prediction can be determined by minimising frustration only [27]. When entropy is dominant, microstructures can be predicted by modelling via Gaussian random waves [29]. Absolute scaling of the scattering curves is essential, as was demonstrated already for micelles in the pioneering work of Hayter and Penfold, performed on D11 and D17 at ILL more than 40 years ago [30]. Using absolute scaling, the variation of effective curvature of ionic micelles versus surface dissociation allowed to confirm the dressed model of micelles [31].

The scattering experiment always allows to determine a mean spacing D* (expressed in nm) from the peak position, and the known area per surfactant molecule allows to measure the total area of contact between water and oil per volume of sample (specific interfacial area per volume *Σ*, expressed in nm^−1^). The product of the two is a dimensionless quantity that varies with the polar volume fraction. The corresponding swelling curve (ΣD^*^ versus *ϕ*_apolar_) can be compared to predictions of all parameter-free models of microemulsions. Therefore, performing scattering experiments at several volume fractions and on absolute scale (needed for determining *Σ*), either in SAXS or in SANS, allows to prove, which microstructure is present in any microemulsion using the dimensionless quantity ΣD^*^. Oppositely, when the swelling law is not met, the corresponding microstructure is disproven. Only the original Talmon-Prager model [32] does not predict the presence of a maximum in the scattering, and this situation has never been found experimentally. The delicate point is that near the symmetry point, most models except the high internal phase microemulsions (HIPME) [33] give very similar peak positions [34].

1 $${D}^{*}\Sigma \approx 3$$

This means that studying a microemulsion close to 0.5 is only relevant to determine a typical size via a three-parameter fit, with results that can be easily calculated a priori if interfacial film volume and thickness are known. However, this does not allow to determine the type of microstructure, unless the *q*-range is extended to large values (*qR* > 15) in order to allow indirect Fourier transformation [35] (but experimentally that would also be problematic, as that *q*-range is susceptible to scattering arising from local structuring). In order to discriminate between totally flexible random bicontinuous and locally cylindrical structures, volume fractions close to 25% must be measured [36]. Measuring only close to 50% and not taking absolute intensities is a waste of scattering time if the local microstructure is not known beforehand and compatible with the phase diagram [37].

## Results and discussion

### Phase diagram

**Fig. 1:**
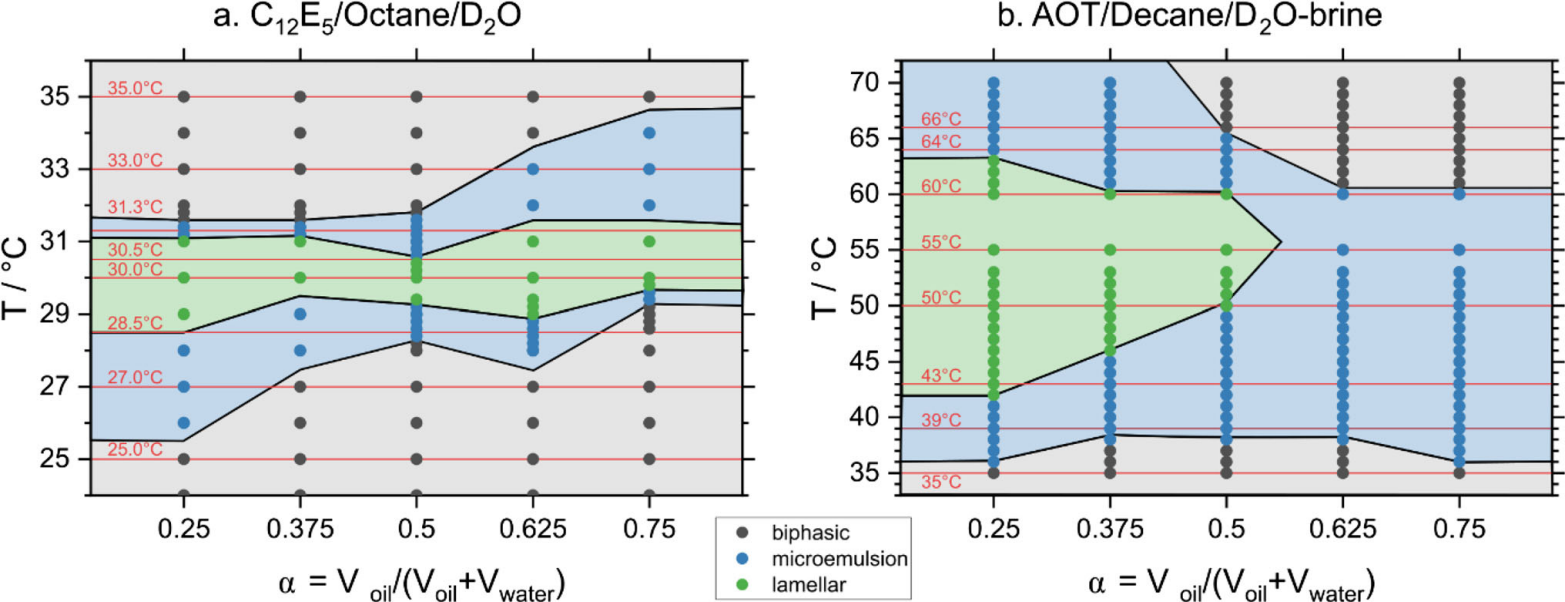
Phase diagram of the two studied microemulsion systems: C_12_E_5_/octane/D_2_O and AOT/decane/D_2_O-brine (0.4 wt.% NaCl) for a constant surfactant concentration of 7 or 12 wt.%, respectively, as a function of volume mixing ratio α of oil/water(brine) and temperature. The temperatures that were chosen for SANS measurements are shown with horizontal dotted red lines

In a first step, we carefully determined the phase behaviour of the two different microemulsion systems for fixed surfactant concentrations of 7 or 12 wt.% for C_12_E_5_ and AOT, respectively, as a function of volume mixing ratio *α* of oil/water(brine) and temperature. The phase behaviour is shown in Fig. 1 and demonstrates that there is a single-phase microemulsion region in both cases that goes all the way from the pure water side to the pure oil side. For the C_12_E_5_ system, one finds also a lamellar phase that stretches through the whole centre of the microemulsion channel, while for the AOT system a lamellar phase is only seen for the water-rich side of the phase diagram, and it also occurs in the centre of the microemulsion phase. It might be noted that compared to the published phase diagram of C_12_E_5_/octane/water [11], our phase diagram is shifted to lower temperature by about 2 °C, which may be attributed to using D_2_O instead of H_2_O. Also, we have observed a lamellar phase at all values of *α*, which could be attributed to the fact that we studied here only at a spacing of 0.125 in *α*, as used subsequently in the SANS experiments. It might be noted that for the AOT system, the salt concentration was decreased from 0.6 wt.% (used in [14]) to 0.4 wt.% in order to have a convenient temperature window for our experiments. Interestingly, we observe also that the lamellar phase is somewhat more extended than in that publication, even going to values larger than *α* = 0.5.

### SANS

**Fig. 2:**
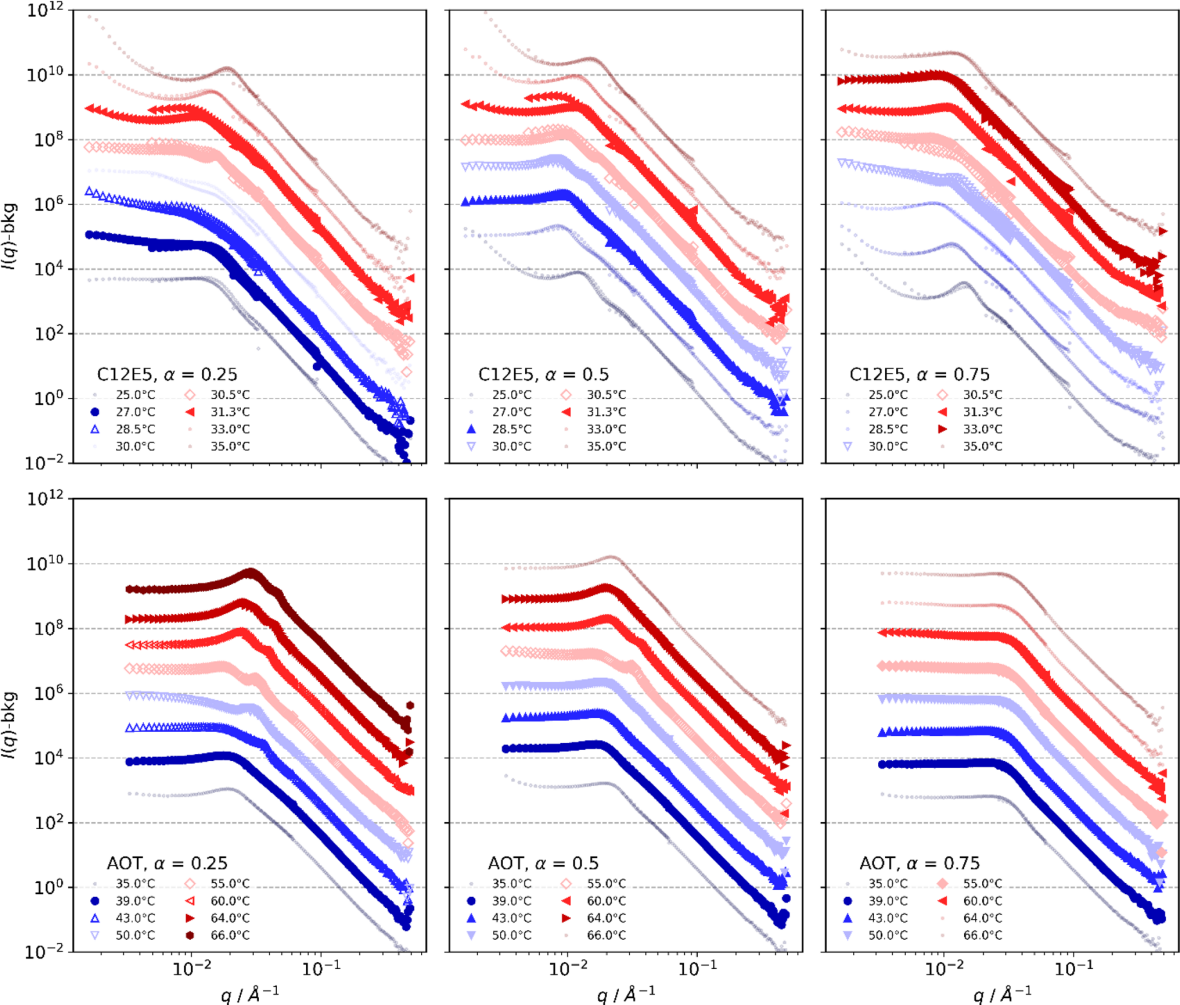
SANS spectra for the C_12_E_5_ (top row) and the AOT (bottom row) microemulsion for three different volume mixing ratios α of oil/water(brine) (0.25, 0.5, 0.75) measured at different temperatures. Samples that are microemulsions are shown using filled symbols and lamellar samples with open symbols. Biphasic samples are included as smaller circles. The spectra are shifted by *n* decades to ensure readability, where *n* = 0 … 7 for increasing temperatures

For obtaining detailed structural information, SANS experiments were done over the whole range of *α* from 0.25 to 0.75 in steps of 0.125 and varying systematically the temperature. The results for three of the five oil-to-water ratios are shown in Fig. 2 (the remaining two are shown in the supporting material in Figure S1). Samples in the microemulsion phase show a more or less marked correlation peak, while for samples in the lamellar phase this peak is more pronounced and often a second-order peak is seen, indicating longer-range order. Samples in the biphasic region typically show an increasing intensity towards low-*q*, arising from the ongoing phase separation. In general, the peaks are more pronounced for the AOT case, which is to be expected simply as the surfactant concentration here is substantially higher, and correspondingly, the structural domains are more densely packed. Beyond the correlation peak, the scattering curves follow a *q*^−4^ Porod behaviour. Deviations at high-*q* are larger for the C_12_E_5_ system, since the EO head groups lead to a less sharp interface between the two sub-phases. However, it can also be noted that some of the AOT microemulsions at lower *α*-values up to 0.5 show a second peak, which likely indicates the presence of a droplet structure. Interestingly, it appears only at higher temperatures and for *α* = 0.375 and 0.5 only for the temperature closest to the lamellar phase. Accordingly, there seems to be some relation to the presence of the lamellar phase but this second peak does not come at twice the q-value of the first peak (especially not for the lowest α value), which speaks against the presence of a lamellar structure.

For a quantitative analysis, the SANS data were analysed with the help of the Teubner-Strey model [17] that typically yields a good description of scattering curves of bicontinuous microemulsions but is also able to describe the spectra of droplet microemulsions [38]. The scattering intensity in the Teubner-Strey model is given in Eq. (2), and the two parameters of this model describe the mean spacing *d* of the domains and the correlation length *ξ* (characteristic domain size):

2 $$I(q)=\frac{8\pi {\Delta SLD}^{2}\phi \left(1-\phi \right)/\xi }{{a}^{2}-2b{q}^{2}+{q}^{4}}$$

where $${a}^{2}={\left({\left(\frac{2\pi }{d}\right)}^{2}+\frac{1}{{\xi }^{2}}\right)}^{2}$$ and $$b={\left(\frac{2\pi }{d}\right)}^{2}+\frac{1}{{\xi }^{2}}.$$

**Fig. 3:**
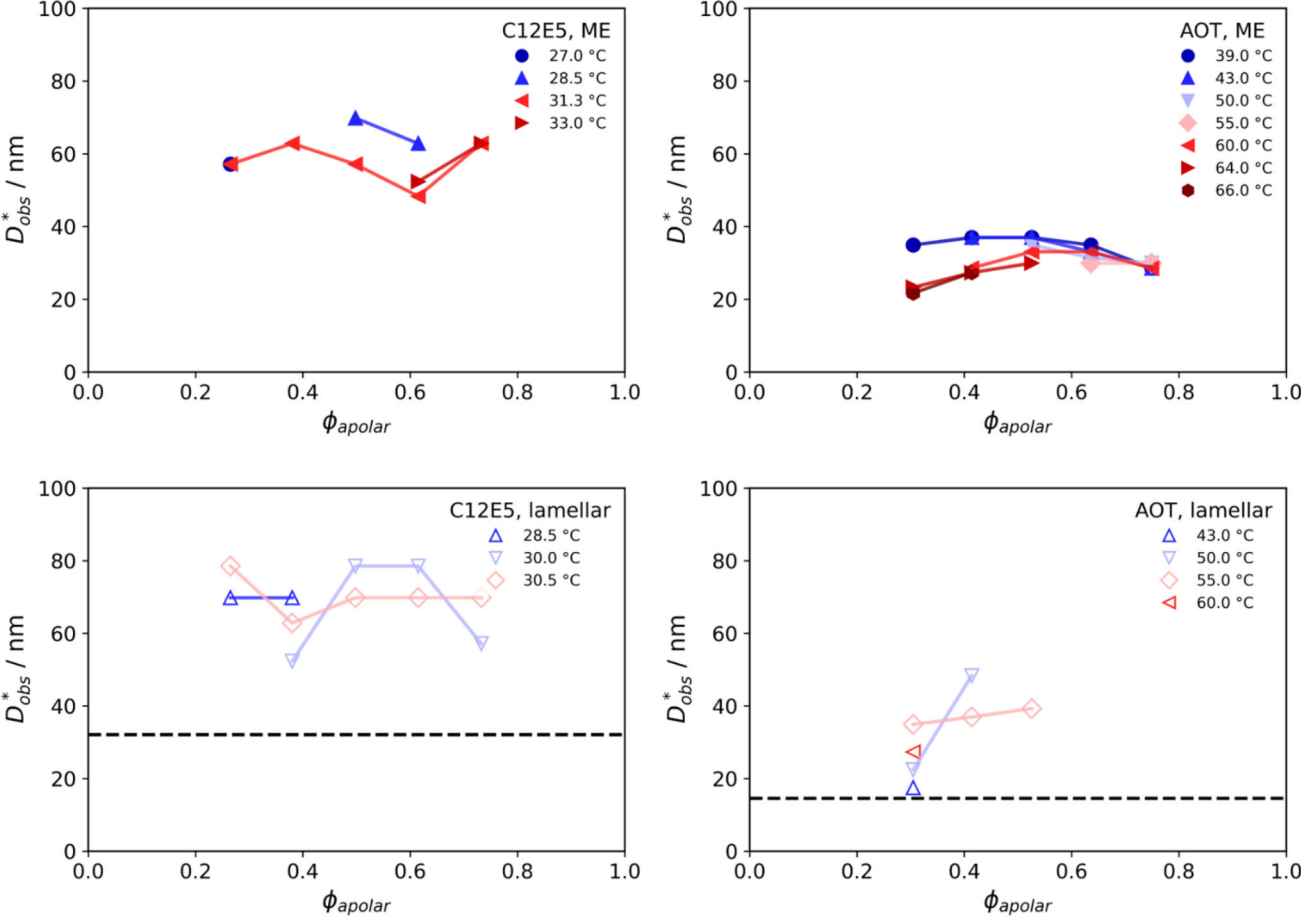
Mean spacing D_obs_ (from the SANS data) for the C_12_E_5_ (left) and the AOT (right) microemulsions (top) and lamellar phases (bottom) as a function of the apolar volume fraction *ϕ*_apolar_, measured at different temperatures. The dotted lines in the lamellar plots indicate the predicted spacing if all surfactant was contained in a flat bilayer

The fits are shown in the supporting material in Figure S2. The fit works well in some cases but did not yield reliable results for the mean spacing in all cases. This can be at least partially attributed to the presence of a low-*q* upturn, which disturbs the fit in the relevant *q*-range. Thus, the observed mean spacing $D_{{{\text{obs}}}}^{*}$ was determined from the approximate intensity peak location *q*_max_ using the Bragg equation D^*^_obs_ = 2π/*q*_max_. $D_{{{\text{obs}}}}^{*}$ is given in Fig. 3 as a function of the apolar volume fraction *ϕ*_apolar_ for various temperatures. This value changes only little for the C_12_E_5_ system and decreases somewhat with *ϕ*_apolar_ for low temperatures and increases somewhat for high temperature. In contrast, for the AOT system it goes over a maximum at around equal polar and apolar volume fractions (*ϕ*_apolar_ = 0.5), being generally larger at lower temperature. In general, the values for $D_{{{\text{obs}}}}^{*}$ are much larger for the C_12_E_5_ microemulsion, which simply arises from the fact that here the surfactant concentration is much lower, and in addition, it has the smaller head group. In the case of the lamellar samples, we can readily calculate the predicted spacing for a completely flat bilayer through D* = 2* l*/*ϕ*_s_, where *l* is the length of a surfactant molecule (half the bilayer thickness) and *ϕ*_s_ is the volume fraction of surfactant. The length *l* = *V*_m_/*a*_0_ can be estimated using the molecular volume *V*_m_ and the head group area *a*_0_. The latter is taken to be roughly 60 Å^2^ and 80 Å^2^ for C_12_E_5_ and AOT, respectively. For both surfactants, $D_{{{\text{obs}}}}^{*}$ is considerably larger than the predicted value for a flat bilayer, which indicates that large amounts of surface area are lost in fluctuations and defects. This “missing surface” is treated as an adjustable amphiphilic factor in the standard flexible model and can be due to solubilisation of the surfactant outside of the interface, surface undulation with short wavelength of the film, or molecular roughness. In our mind, this is one of the most important open problems in quantitative understanding of real microemulsions.

The second key geometric parameter describing the structure of the microemulsions is the total specific surface area/volume *Σ* = *c*_s_*a*_0_, where *c*_s_ is the number concentration of surfactant molecules. In theory, *Σ* can be determined from the Porod limit of the SANS spectra. Porod’s law is given by [39]:

3 $$I\left(q\to \infty \right)=\frac{P}{{q}^{-4}}+bkg$$

and was fitted to the experimental data for 0.16 < $q$  < 0.46. Here, $P$ is the Porod constant and *bkg* is the incoherent background scattering intensity. The Porod constant is related to *Σ* through

4 $$P=\frac{\Sigma }{2\pi\Delta SL{D}^{2}}$$

where *ΔSLD* is the scattering length density difference. We can eliminate the dependence on Δ*SLD* using the scattering invariant *Q*_inv_, which is given by

5 $${Q}_{\mathrm{inv}}={\int }_{0}^{\infty }I\left(q\right){q}^{2}\mathrm{d}q= 2{\pi }^{2}{\phi }_{\mathrm{apolar}}\left(1-{\phi }_{\mathrm{apolar}}\right)\Delta SL{D}^{2}$$

The integral in Eq. (5) can be determined by numerical integration of the scattering data (for details, see supporting material). Using Eqs. (4) and (5), we find that

6 $$\Sigma =\frac{P}{{Q}_{\mathrm{inv}}}\pi {\phi }_{\mathrm{apolar}}\left(1-{\phi }_{\mathrm{apolar}}\right)$$

which is now independent of Δ*SLD*. In the case of C_12_E_5_, the interface between oil and water domains is thick and heterogeneous due to the bulky ethoxy head group. The internal structuring within the bilayer introduces additional scattering intensity. For this reason, a determination of *Σ* according to Eq. (6) is impossible for C_12_E_5_ and leads to erroneous values. Instead, we have opted to estimate the specific area/volume by *Σ* = *c*_s_*a*_0_, using a head group area of *a*_0_ = 60 Å^2^ [40]. For AOT, the determination of *Σ* using the procedure outlined above works well, due to the sharp oil–brine interface.

**Fig. 4:**
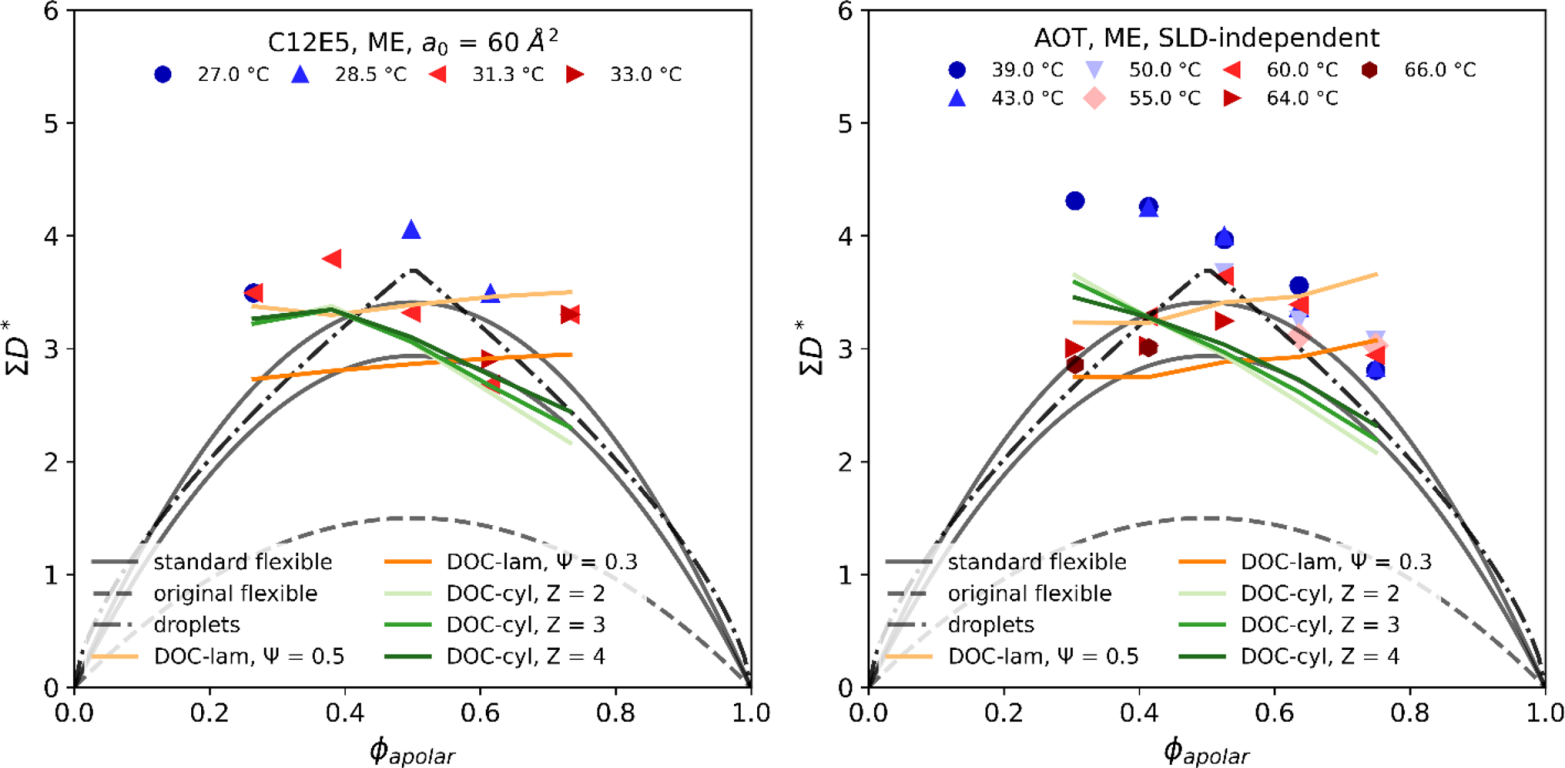
Dimensionless product of mean spacing D^*^_obs_ and specific area/volume *Σ* for the C_12_E_5_ (left) and the AOT (right) microemulsion as a function of the apolar volume fraction *ϕ*_apolar_ measured at different temperatures. For C_12_E_5_, *Σ* was estimated assuming a constant area per molecule *a*_0_ of 60 Å^2^, while for AOT, *Σ* was determined from the scattering invariant and the Porod constant. For comparison, different theoretical model predictions are indicated by lines

Multiplying *Σ* with the mean spacing $D_{{{\text{obs}}}}^{*}$ yields a dimensionless parameter, which is characteristic for the geometry of the present structure. This parameter is shown in Fig. 4 and contains the most important findings of this work.

The dimensionless quantity ΣD^*^ can be compared to predictions of various models describing the mesoscopic structure of microemulsions [34].

The simplest case to consider are spherical W/O droplets without coalescence. Since the droplets repel and do not coalesce, Chen et al*.* have shown that the expected mean spacing D^*^ (derived from the scattering peak position *q*_max_ via D* = 2π/*q*_max_) can be approximated at typically 1% precision to [41]:

7 $${D}^{*}=1.225 {n}^{-\frac{1}{3}}$$

where the droplet (Voronoi centre) number density *n* is fixed through the volume fraction of the dispersed phase $\phi =n\frac{4}{3}\pi {R}^{3}$ and the specific surface/volume $\Sigma =n4\pi {R}^{2}$, *R* being the droplet radius [42]. For the dimensionless product ΣD^*^, we find:

8 $$\Sigma {D}^{*}=5.9{\phi }^{\frac{2}{3}}$$

For flexible interfaces, the dimensionless product for bicontinuous microemulsions varies according to [43]:

9 $$\Sigma {D}^{*}=6\phi \left(1-\phi \right),$$

which we will refer to as the original flexible model. However, this prediction was never found experimentally, because it does not take effective repulsion between local droplets into account. Considering alternation between water and oil domains introduces a factor of 2 in Eq. (9) to give:

10 $$\Sigma {D}^{*}=2\beta \phi (1-\phi )$$

where $\beta$ is a numerical factor in the range of 5.87–6.82 [1, 38]. Equation (10) is often referred to as the standard flexible model.

The construction of a DOC model starts by placing Poisson points randomly in space [27]. The density of points *n* is connected to D^*^ through the scattering peak position by D^*^ = *n*^−1/3^ = 2π/*q*_max_. Depending on the spontaneous curvature of the surfactant film, we can distinguish between two cases. In cases where the radius of curvature is small compared to the distance between neighbouring Poisson points, we approximate the bicontinuous structure by a network of spheres that are connected by an average of Z cylinders per sphere (DOC-cylinder). The choice of Z results in various different structures. For example, Z = 2 resembles wormlike micelles, while Z = 3 and Z = 4 resemble a “molten” cubic lyotropic liquid crystal structure. The initial DOC-cylinder model [44] considered all possible connections between cylinders from giant wormlike micelles to molten cubic liquid crystals. A subset of this model considering only the connectivity Z = 2 but introducing a nonzero temperature was published by Tlusty et al. [45]. There are no explicit predictions of peak positions but the result is close to the DOC-cylinder case with Z = 2. Since none of the investigated microemulsions was described by the more general DOC-cylinder model, the Tlusty model cannot be tested with our systems.

In cases where the curvature is large compared to *n*, we approximate the structure by a set of connected pieces of bilayer (DOC-lamellar). This construction resembles a tightly folded thick sheet of bilayer and is water-continuous and thus conducting. To avoid regions of high curvature, the oil is divided into two pseudo-phases of volume fractions *Ψ* and 1-*Ψ* and the bilayer between regions of the same pseudo-phase is removed. For the case of *Ψ* = 0.5, the DOC-lamellar model reduces to the symmetric sponge phase. A more detailed description of these models designed for stiff microemulsions that satisfy volume, surface, and curvature constraints simultaneously can be found in ref [27]. Extending this to flexible microemulsions has been done by Roux et al. [46] in the case of locally lamellar structures with infinitely thin films but introducing temperature. The expression for the scattering peak position is exactly the same than for the standard flexible model and is not discussed separately in this work.

Looking at Fig. 4, we find that the C_12_E_5_ data are best described by the symmetric sponge DOC-lamellar model with *ψ* = 0.5, especially at higher temperature. For AOT, we have to discriminate between temperatures that are below and above the phase transition temperature of around 55 °C (see phase diagram in Fig. 1). Below 55 °C, the only structural model that predicts the correct shape of the swelling curve are connected cylinders. Above 55 °C, the standard flexible model and the molten liquid crystal DOC cylinders are both compatible with the observation.

Most of the swelling studies published so far were done at 50% volume fraction, for which all values degenerate and no information can be extracted. At 75% and 25% volume fraction, predictive structural models can be discriminated by experiments. One of the most precise swelling plots was established in the case of extracting molecules, such as TBP, that are crucial in nuclear fuel recycling. These form microemulsions in the presence of co-surfactants and diluent modifiers. The standard flexible model of microemulsions by De Gennes–Taupin was clearly discriminated. Most microemulsions with asymmetric salts tested were best modelled as connected cylinders. Upon addition of lithium chloride, a very cosmotropic salt, a structure close to a locally lamellar microstructure (similar to what is sometimes named HIPME) was the only one compatible with the observed scattering [47]. In the case of microemulsions with a liquid ionic surfactant and room temperature ionic liquids as polar pseudo-phase, no currently available model of microemulsion structure was compatible with the swelling experiment results [48]. Even after 50 years of measurements of SAXS/SANS on absolute scale, there are still a large number of unknown molecular mechanisms leading to differences in microstructure to discover.

### Conductivity

**Fig. 5:**
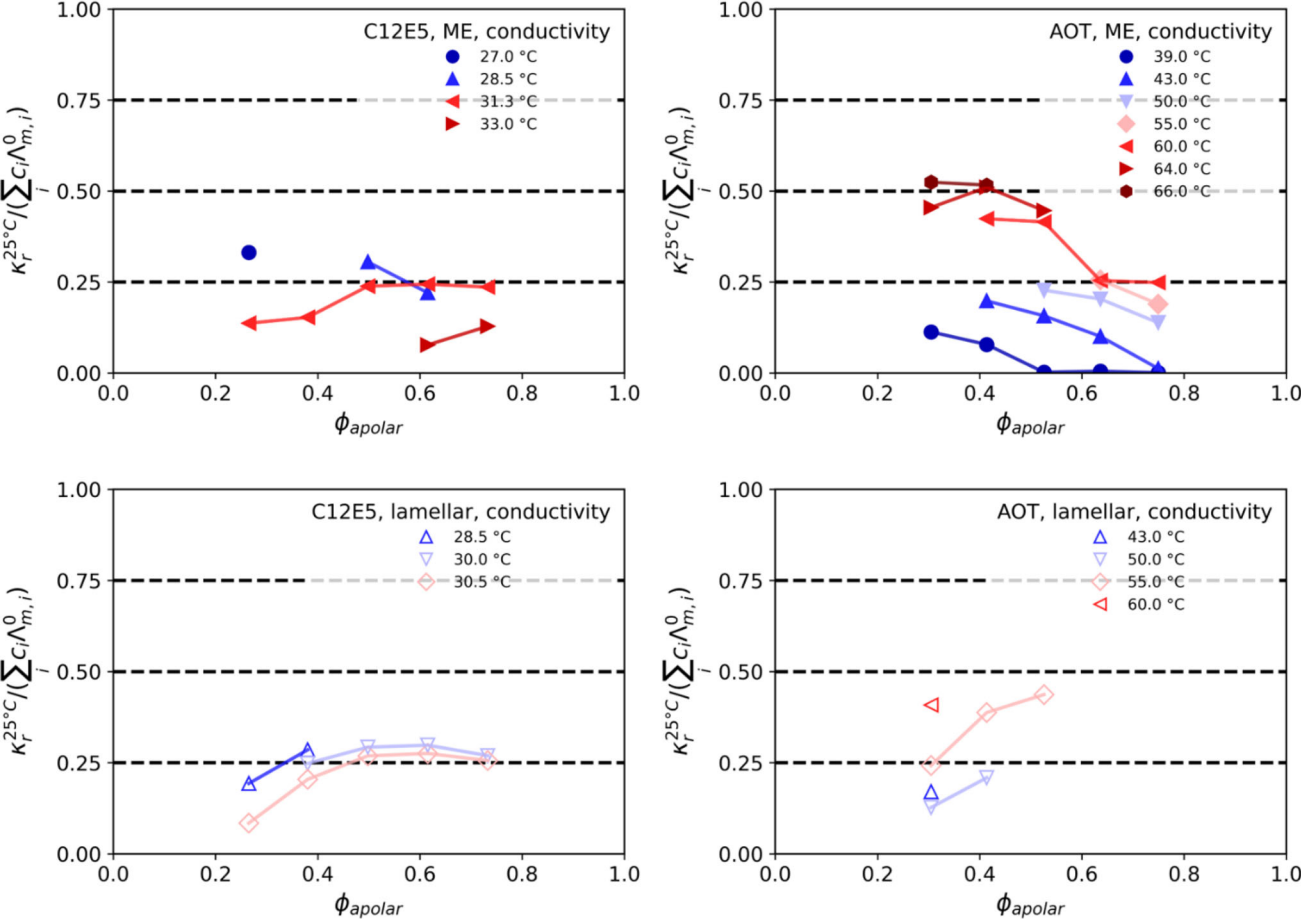
Dimensionless conductivity for a constant surfactant concentration of 7 or 12 wt.% of C_12_E_5_ or AOT, respectively, as a function of the apolar volume fraction *ϕ*_apolar_, measured at different temperatures. Upper row: microemulsions. Lower row: lamellar phase

To gain further structural insight, we measured the conductivity of the different microemulsion systems at different temperatures and varying the volume mixing ratio α of oil/water(brine) in the same way as in the SANS experiments. The experimentally obtained values were corrected for the expected temperature effect assuming a linear correlation according to

15 $${\kappa }^{25^\circ \mathrm{C}}=\frac{\kappa }{1+f\cdot (T-{T}_{\mathrm{ref}})/100\%}$$

where $\kappa$ is the measured conductivity, $f$ is a temperature correction coefficient, here assumed to be 2%/K [49], T is the current temperature and ${T}_{\mathrm{ref}}$ is 298 K. In addition, they were further normalised by dividing by the volume fraction of the conducting phase ${\phi }_{\mathrm{cond}}$ (for C_12_E_5_ samples: D_2_O; for AOT samples: D_2_O + AOT head) to give ${\kappa }_{r}^{25^\circ \mathrm{C}}={\kappa }^{25^\circ \mathrm{C}}/{\phi }_{\mathrm{cond}}$ (shown in supporting material in Figure S6). To achieve comparability between the two systems, ${\kappa }_{r}^{25^\circ \mathrm{C}}$ was further rescaled by dividing by the sum of the products of the molar conductivity ${\Lambda }_{m,i}$ and the molar concentration ${c}_{i}$ of the conducting ions in the sample. For C_12_E_5_, this includes the Na^+^ and Cl^−^ ions from the 5 mM D_2_O-brine that was used for the conductivity measurements. For the AOT samples, the surfactant head group is assumed to be fully dissociated into Na^+^ and the bulky surfactant anion. If we neglect the surfactant anions, the conductivity is given by the Na^+^ and Cl^−^ ions from the 0.4 wt.% D_2_O-brine and the Na^+^ from the head group. This final dimensionless conductivity is shown in Fig. 5 and gives the relative value of conductivity compared to that of fully freely moving ions in the system. It might be noted that for a balanced perfect sponge structure here a value of 0.67 would be expected, but Anderson and Wennerström computed the “obstruction factor” via the comparison of diffusion coefficients in neighbouring lyotropic liquid crystalline phases [50]. They found that in the DOC-cylinder regime, the obstruction factor is 2/3; in the DOC-lamellar phase, it is lower (with no exact value given, but close to 1/2); however, in the case of missing surface due to fluctuations such as obtained with AOT, they found that the obstruction factor is much higher than these values; i.e. there is less obstruction for flexible microemulsions.

The data are given in Fig. 5 and show the interesting observation that for the C_12_E_5_ system the dimensionless conductivity is generally higher at low temperature: this means that the cations *desorb* from the interface at higher temperature: the head group is dehydrated and therefore less favourable to host cations by complexation [51]. The reduced conductivity is less than one half, which means that most of the cations of the background salt are complexed by ethoxy, while some of the anions in the counter-ion cloud are slowed down in the head-group area.

For the AOT-based system, exactly the opposite temperature behaviour is observed: the conductivity at high temperature comes close to the expected value in bulk water. The counter-ions move and are hydrated. Increase in temperature means less binding of the Na^+^, which is acting like a “free” counter-ion, while it decreases to almost zero at the lower temperatures. This means that at low temperatures, a large fraction of the Na^+^ ions are bound in the Stern layer.

**Fig. 6:**
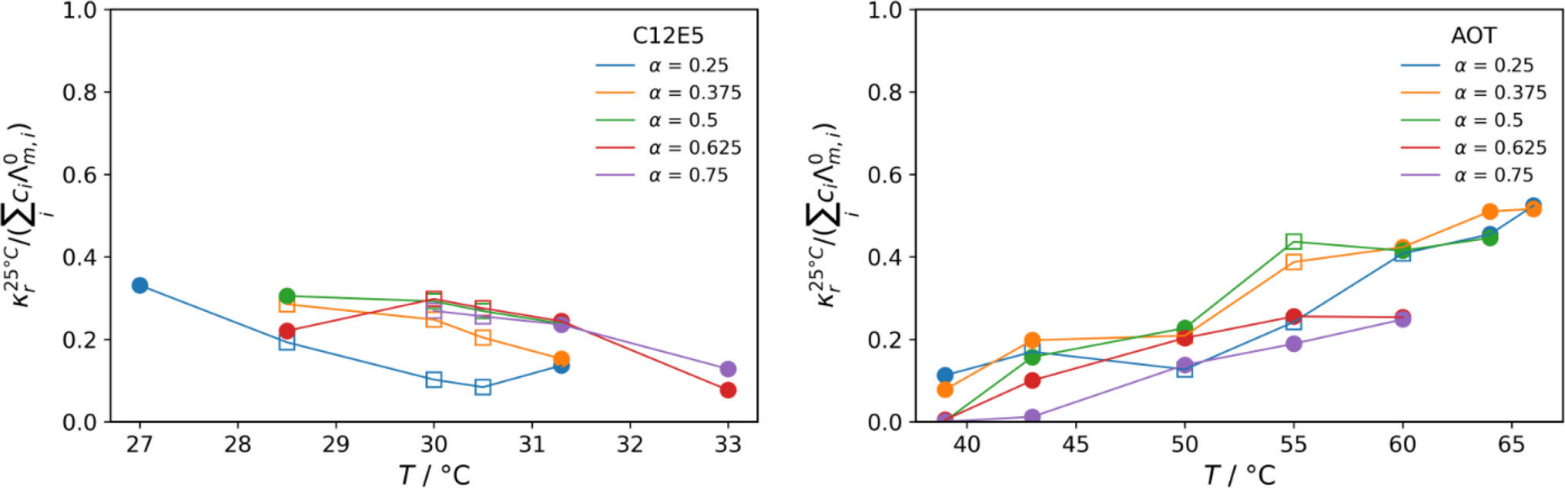
Dimensionless conductivity for constant surfactant concentrations of 7 and 12 wt.% for C12E5 and AOT, respectively, as a function of temperature. Biphasic samples are shown with half-filled triangles, microemulsions samples with filled spheres and lamellar samples with open squares

The temperature dependence is shown more directly in Fig. 6. Here one can see a temperature-induced decrease of tortuosity, a premise of percolation. The reduced conductivity decreases smoothly, but not as a power law. One observes a drastic increase in conductivity with increasing concentration of the polar part [52] or as a function of temperature [53]. Accordingly, here we see also substantial differences of the C_12_E_5_ and the AOT system, where in a similar range of volume fractions the former always forms a water-continuous structure, while the latter transits to disconnected aqueous domains. This can be compared to NMR data on a similar C_12_E_5_ system (exchanging octane by tetradecane) that shows that the self-diffusion coefficient decreases continuously with increasing oil content [7]. This is similar to our observation at lower temperature. Interestingly, we see the opposite trend for T > 30 °C. This could indicate the formation of a more connected structure, but could also potentially result from a reduced binding of the ions, especially the Na^+^ ions, to the ethoxy head groups.

## Conclusion

Fifty years after the first pioneering SANS studies of microemulsions, we have shown that microemulsion peaks can not only be fitted by adjusting three parameters to reproduce any broad “bump”. Instead, we consider that any microemulsion structure can be compared to models, when both the mean spacing and the total surface per volume are taken into account simultaneously. The Cates-Andelmann-de Gennes model analysis gives the right peak at 50% volume fraction, just as all other available models do. Only the initial Talmon-Prager model does not predict a peak [34]. Using the swelling plot, and considering points far away from the symmetry degeneracy at 50%, the locally lamellar model yields a better prediction for the C_12_E_5_ microemulsions than the standard flexible model. In the case of the ionic AOT microemulsions, the standard flexible model is the best at low water content, where charging of the interface by counter-ion dissociation becomes important. Using the same methodology, we think that a large number of microemulsion microstructures structures have not yet been investigated in a domain large enough by varying the polar volume fraction. The most advanced model including molecular sizes and interactions is due to Gompper and Goos [54]. Explicit scattering predictions have been derived from this theory. This is the most predictive model known so far: excellent fits have been shown with 6 parameters all having a clear physical meaning [24, 55]. We did not test this model since we are only focussing on the peak position.

To the best of our knowledge, even after 50 years, there is no predictive theory of surfactant film bending that can predict the scattering measured in the so-called film contrast. The scattering in film contrast can only be fitted and parametrised, without any information gain. The story of understanding USWANS (and USWAXS) of different microemulsions that are used in home care and pharmacy is far from being finished in 2023.

### Supplementary Information

Below is the link to the electronic supplementary material.

Supplementary file1 (PDF 901 kb)

## Data Availability

Raw SANS data were generated at Institut Laue-Langevin (Grenoble, France). Additional information about the data can be accessed through the data DOI https://doi.org/10.5291/ILL-DATA.9-10-1704.

## References

[1] Gradzielski M, Duvail M, de Molina PM, Simon M, Talmon Y, Zemb T (2021). Chem. Rev..

[2] Szleifer I, Kramer D, Ben-Shaul A, Gelbart WM, Safran SA (1990). J. Chem. Phys..

[3] Ishikawa K, Behrens M, Eriksson S, Topgaard D, Olsson U, Wennerström H (2016). J. Phys. Chem. B.

[4] Dufrêche JF, Zemb Th (2020). Curr. Opin. Colloid Interface Sci..

[5] Hyde ST, Barnes IS, Ninham BW (1990). Langmuir.

[6] Bodet JF, Bellare JR, Davis HT, Scriven LE, Miller WG (1988). J. Phys. Chem..

[7] Olsson U, Shinoda K, Lindman B (1986). J. Phys. Chem..

[8] Lichterfeld F, Schmeling T, Strey R (1986). J. Phys. Chem..

[9] Guering P, Lindman B (1985). Langmuir.

[10] de Gennes PG, Taupin C (1982). J. Phys. Chem..

[11] Cates ME, Andelman D, Safran SA, Roux D (1988). Langmuir.

[12] Barnes IS, Hyde ST, Ninham BW, Derian PJ, Drifford M, Zemb TN (1988). J. Phys. Chem..

[13] Kahlweit M, Strey R, Haase D, Kunieda H, Schmeling T, Faulhaber B, Borkovec M, Eicke H-F, Busse G, Eggers F, Funck Th, Richmann H, Magid L, Söderman O, Stilbs P, Winkler J, Dittrich A, Jahn W (1987). J. Colloid. Interface Sci..

[14] Chen S-H, Chang S-L, Strey R (1990). J. Chem. Phys..

[15] Gradzielski M, Schmidt RF, Simon M, Malo de Molina P, Zemb T, Prevost S (2020). Institut. Laue-Langevin (ILL).

[16] C. Dewhurst, GRASP: Graphical Reduction and Analysis SANS Program for Matlab (2003).

[17] Teubner M, Strey R (1987). J. Chem. Phys..

[18] Schubert K-V, Strey R (1991). J. Chem. Phys..

[19] Pieruschka P, Safran SA (1995). Europhys. Lett..

[20] Arleth L, Marc̆elja S, Zemb T (2001). J. Chem. Phys..

[21] Duvail M, Arleth L, Zemb T, Dufrêche J-F (2014). J. Chem. Phys..

[22] Duvail M, Dufrêche J-F, Arleth L, Zemb T (2013). Phys. Chem. Chem. Phys..

[23] Gompper G, Endo H, Mihailescu M, Allgaier J, Monkenbusch M, Richter D, Jakobs B, Sottmann T, Strey R (2001). Europhys. Lett..

[24] Peltomäki M, Gompper G, Kroll DM (2012). J. Chem. Phys..

[25] Choi SM, Chen SH, Sottmann T, Strey R (2002). Physica A.

[26] Kirste R, Porod G (1962). Kolloid-Zeitschrift Und Zeitschrift Für Polymere.

[27] Zemb TN (1997). Colloids Surf. A Physicochem. Eng. Asp..

[28] Barnes IS, Zemb TN (1988). J. Appl. Crystallogr..

[29] Pieruschka P, Marčelja S (1992). J. Phys. II France.

[30] Hayter JB, Penfold J (1983). Colloid. Polym. Sci..

[31] Hayter JB (1992). Langmuir.

[32] Talmon Y, Prager S (1978). J. Chem. Phys..

[33] Wolf L, Hoffmann H, Teshigawara T, Okamoto T, Talmon Y (2012). J. Phys. Chem. B.

[34] T.N. Zemb, I.S. Barnes, P.-J. Derian, and B.W. Ninham, in *Trends in Colloid and Interface Science IV*, edited by M. Zulauf, P. Lindner, and P. Terech (Steinkopff, Darmstadt, 1990), pp. 20–29. 10.1007/BFb0115518

[35] Strey R, Glatter O, Schubert K-V, Kaler EW (1996). J. Chem. Phys..

[36] Zech O, Bauduin P, Palatzky P, Touraud D, Kunz W (2010). Energy Environ. Sci.

[37] Scattering methods applied to soft condensed matter (European summer school) (1990 : Bombannes, France), T. Zemb, and P. Lindner, *Neutron, X-Rays and Light. Scattering Methods Applied to Soft Condensed Matter* (North-Holland, Oxford, England, 2002).

[38] Prévost S, Gradzielski M, Zemb T (2017). Adv. Colloid Interface Sci..

[39] Porod G (1951). Kolloid-Zeitschrift.

[40] Sottmann T, Strey R, Chen S-H (1997). J. Chem. Phys..

[41] Chen SH, Sheu EY, Kalus J, Hoffman H (1988). J. Appl. Crystallogr..

[42] Pileni M-P, Zemb T, Petit C (1985). Chem. Phys. Lett..

[43] Binks BP, Meunier J, Abillon O, Langevin D (1989). Langmuir.

[44] Ninham BW, Barnes IS, Hyde ST, Derian P-J, Zemb TN (1987). Europhys. Lett..

[45] Tlusty T, Safran SA, Menes R, Strey R (1997). Phys. Rev. Lett..

[46] Roux D, Coulon C, Cates ME (1992). J. Phys. Chem..

[47] Bauer C, Bauduin P, Diat O, Zemb T (2011). Langmuir.

[48] Zech O, Thomaier S, Bauduin P, Rück T, Touraud D, Kunz W (2009). J. Phys. Chem. B.

[49] Barron JJ, Ashton C (2005). TSP.

[50] Anderson DM, Wennerstroem H (1990). J. Phys. Chem..

[51] Liese S, Gensler M, Krysiak S, Schwarzl R, Achazi A, Paulus B, Hugel T, Rabe JP, Netz RR (2017). ACS Nano.

[52] Lagues M, Sauterey C (1980). J. Phys. Chem..

[53] Cametti C, Codastefano P, Tartaglia P, Chen S-H, Rouch J (1992). Phys. Rev. A.

[54] Gompper G, Goos J (1994). Phys. Rev. E.

[55] Gompper G, Schick M (1994). Phys. Rev. E.

