# Supplementary material for: Structure of microemulsions in the continuous phase channel
Source: Eur Phys J E Soft Matter. 2023 Sep 5;46(9):76. doi: 10.1140/epje/s10189-023-00337-z (PMC10480248; doi:10.1140/epje/s10189-023-00337-z)
Supplement: Supplementary file 1 — Supplementary file1 (PDF 901 kb) [file 10189_2023_337_MOESM1_ESM.pdf]

SUPPORTING INFORMATION

Structures of Microemulsions in the Continuous Phase Channel

Robert Franz Schmidt [a], Sylvain Prévost [b], Michael Gradzielski [a], Thomas Zemb [c]

[a] Stranski-Laboratorium für Physikalische und Theoretische Chemie, Institut für Chemie, Technische Universität Berlin, Straße des 17. Juni 124, D-10623 Berlin, Germany

[b] Institut Laue-Langevin, 71 avenue des Martyrs CS 20156, 38042 Grenoble Cedex 9, France

[c] Institut de Chimie Séparative de Marcoule, ICSM UMR 5257 - CEA/CNRS/UM/ENSCM, Marcoule, 30207, France

Scattering spectra

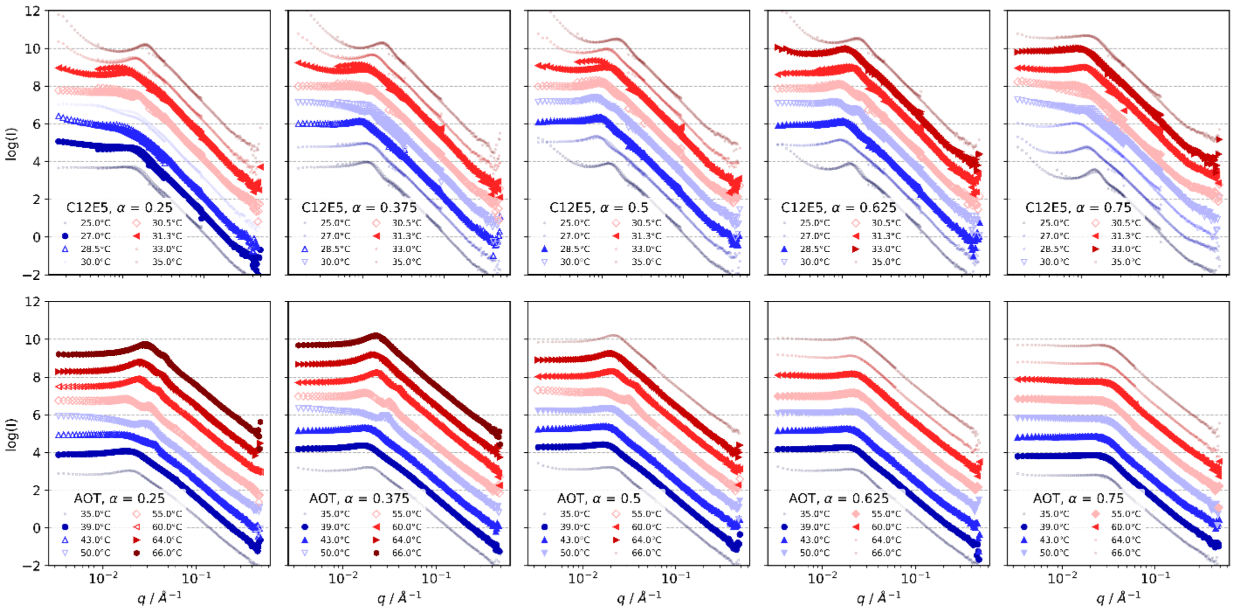

Figure S1: Scattering spectra of all oil/water(brine) mixing ratios for $C_{12}E_5$ (top row) and AOT (bottom row) samples. Samples that are microemulsions are shown using filled symbols, lamellar samples with open symbols. Biphasic samples are included as smaller circles. The spectra are shifted by n decades to ensure readability, where $n = 0 \dots 7$ for increasing temperatures.

Teubner-Strey Fits

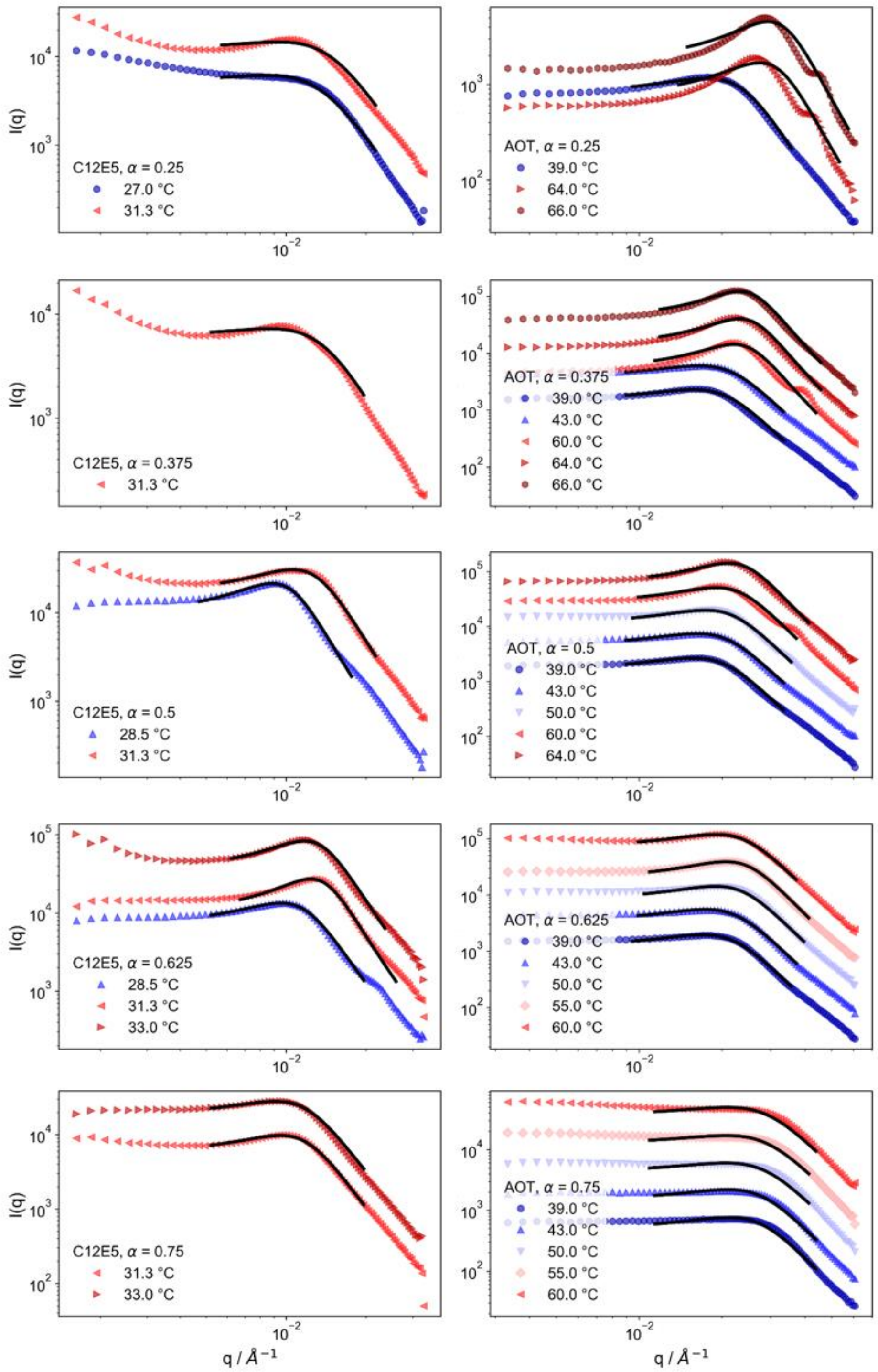

Figure S2: The SANS spectra of the microemulsion samples were fitted with the Teubner-Strey model. The fit range was chosen to be [0.5q_{max}; 2q_{max}], where q_{max} is the presumed location of the scattering peak, which is determined by eye.

Head group area determined through Porod-fitting

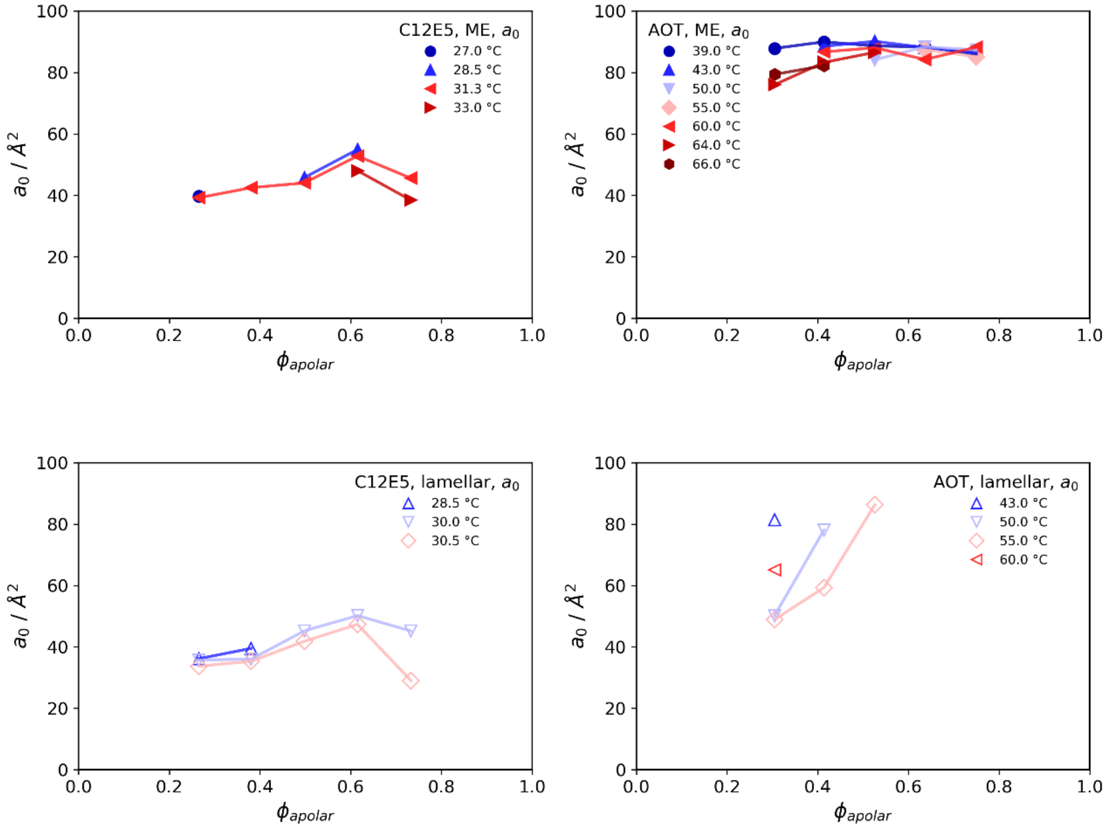

Figure S3: Surfactant head group area as determined from Porod fitting for microemulsion samples (top) and lamellar samples (bottom).

Calculation of SLD

The contrast ΔSLD is calculated as the difference between the SLD of polar and the apolar components

$$\Delta SLD = SLD_{polar} - SLD_{apolar} \qquad \text{(S1)}$$

Which are given by

$$SLD_{polar} = \phi_{polar,D2O} \cdot SLD_{D2O} + \phi_{polar,head} \cdot SLD_{head}$$

$$SLD_{apolar} = \phi_{apolar,oil} \cdot SLD_{oil} + \phi_{apolar,tail} \cdot SLD_{tail} \qquad \text{(S2)}$$

where ϕ_i is the volume fraction of component i within the polar/apolar phase and SLD_i is the scattering length density of component i. The values for SLD_i and a number of other relevant physical parameters are given in Table S1.

Table S1: Relevant physical parameters and scattering length densities. M is the molar mass, ρ is the density and V_m is the molar volume.

	Part	Formula	M / [g/mol]	ρ / [g/cm^3]	V_m / [mL/mol]	SLD [10^{-6} Å^{-1}]
AOT	Overall	$C_{20}H_{27}O_7S$	421.57	1.10	383.25	0.60
	Tail	2 x C_8H_{17} = 3-methylheptane	226.44	0.71	321.19	-0.39
	Head	$C_4H_3O_7S$	195.13	3.14*	62.05	5.70
C12E5	Overall	$C_{22}H_{46}O_6$	406.60	0.96	422.22	0.13
	Tail	$C_{12}H_{25}$ = Dodecane	169.33	0.75	225.92	-0.37
	Head	$C_{10}H_{21}O_6$ = Pentaethylene glycol	237.27	1.13	210.72	0.65
n-octane		C_8H_{18}	114.23	0.70	162.49	-0.52
n-decane		$C_{10}H_{22}$	142.29	0.73	194.91	-0.49
D_2O		D_2O	20.03	1.1	18.04	6.38

$* \rho_{head} = \frac{M_{head}}{V_{head}} = \frac{M_{head}}{\frac{M_{overall}}{V_{m,head}} - \frac{M_{tail}}{\rho_{tail}}}$

Kirste-Porod Correction

According to Kirste and Porod, there is a deviation from Porod's law when q becomes similar to the curvature of interfaces. To remedy this, they proposed an asymptotic expansion of I(q) for large q according to

$$I(q) = 2\pi \Delta SLD^2 \Sigma \cdot \frac{1}{q^4}\left\{1 + \frac{1}{q^2}\left[\frac{1}{4}\langle (C_1 + C_2)^2 \rangle + \frac{1}{8}\langle (C_1 - C_2)^2 \rangle\right]\right\} \quad \text{(S3)}$$

, where C_1 and C_2 are the local principal curvatures of the surface. For fitting, eq. (S3) can be written as

$$I(q) = P \cdot \frac{1}{q^4}\left(1 + C \cdot \frac{1}{q^2}\right) \qquad \text{(S4)}$$

since C_1 and C_2 cannot be accessed individually. Eq. (S4) has been fitted to the experimental data. One example for $C_{12}E_5$ and AOT each are shown in Figure S4.

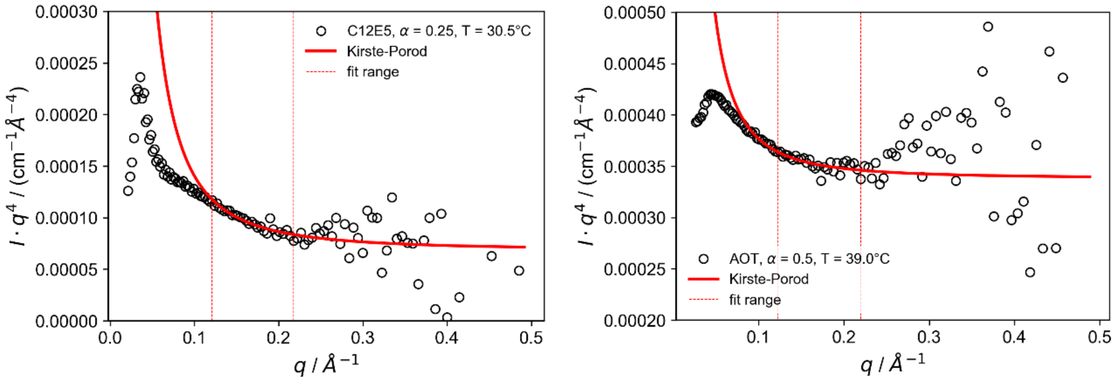

Figure S4: Examples for fits of the Kirste-Porod correction to the experimental data.

If the interfacial film is not homogeneous, the intrinsic scattering at high-q adds up to the scattered intensity in the q-range which is around 0.2 Å^{-1}. If this is the case, making a Kirste-Porod fit leads to unphysical results, because you only add a q^{-2} and a q^{-4} term.

Q-invariant

To obtain the specific area/volume Σ, the Q-invariant Q_{inv} was determined by numerical integration. First, a Guinier fit according to

$$I(q \rightarrow 0) = I(0)e^{-q^2 R_g^2/3} \qquad \text{(S5)}$$

was performed for 2 x 10^{-3} Å^{-1} < q < 7 x 10^{-3} Å^{-1}. The high-q data was fitted using Porod's law (see equation (3) in the main text) for 0.1 Å^{-1} < q < 0.2 Å^{-1}. The fit parameters were then used to predict the low-q behaviour up to 10^{-4} Å^{-1} using Guinier and the high-q behaviour up to 10^{1} Å^{-1} using Porod. The integral in equation (5) in the main text was then obtained by numerical integration using Simpson's rule. The low- and high-q extension is illustrated for one sample in Figure S5.

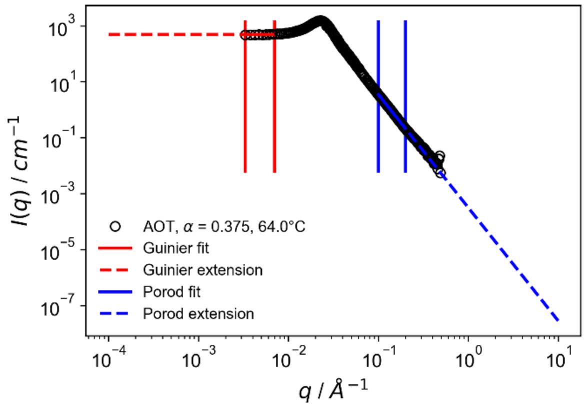

Figure S5: For the determination of Q_{inv}, the low-q behaviour was extrapolated to 10^{-4} $Å^{-1}$ using the Guinier approximation and to 10^1 $Å^{-1}$ using Porod's law. The value for Q_{inv} was then obtain by numerical integration.

Specific conductivity

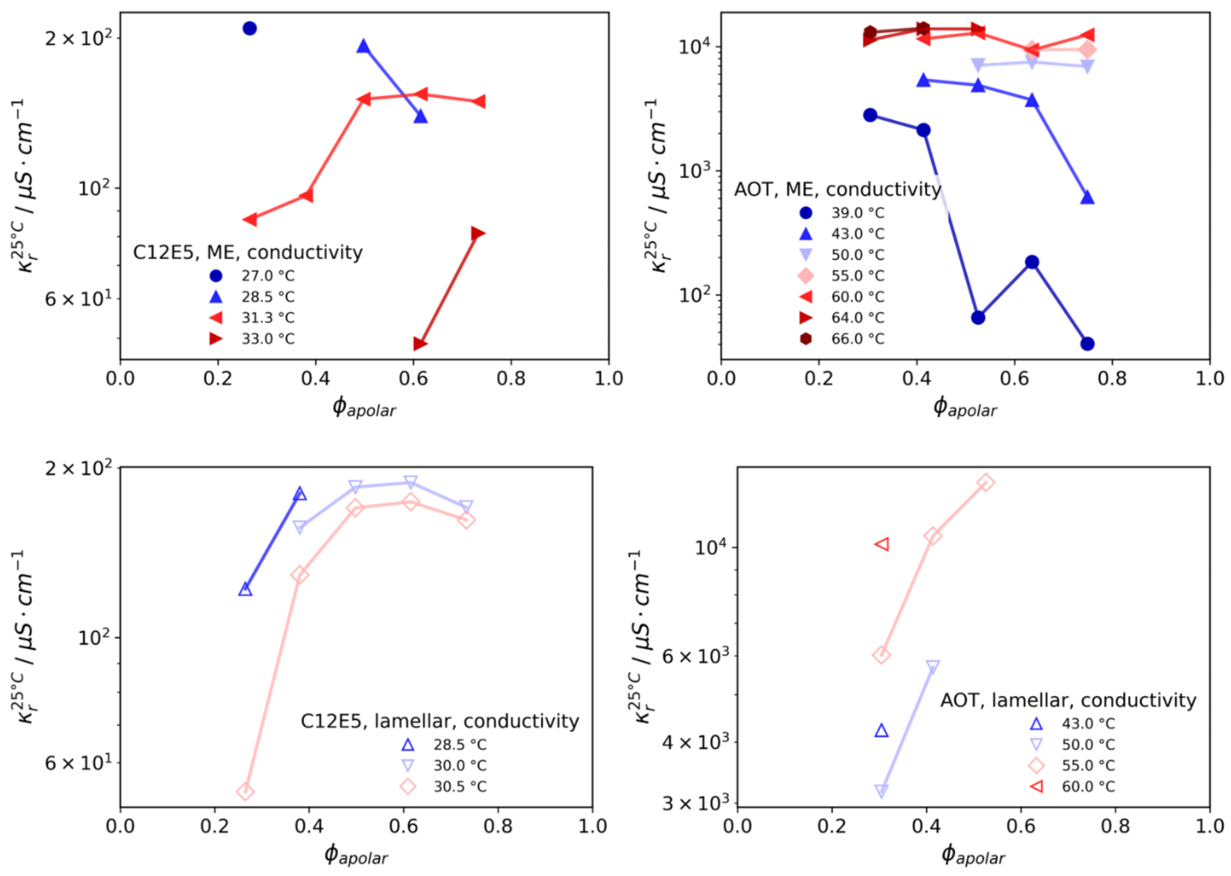

Figure S6: Specific conductivity of the lamellar and microemulsion samples corrected for the volume fraction of the conducting phase.

For C12E5 at high T as well as for AOT at low T, the image as isolated, non-coalescing droplets is very reasonable. In the case of AOT, at low temperatures and low water content, the spontaneous curvature is towards oil, while there is a large fraction of oil. This regime is called the frustrated regime, where there is conflicting terms in the free energy of the effective final structure.
